# Lithuania’s experience in reducing caesarean sections among nulliparas

**DOI:** 10.1186/s12884-018-2052-2

**Published:** 2018-10-25

**Authors:** Justina Kacerauskiene, Meile Minkauskiene, Tahir Mahmood, Egle Bartuseviciene, Dalia R. Railaite, Arnoldas Bartusevicius, Mindaugas Kliucinskas, Ruta J. Nadisauskiene, Kastytis Smigelskas, Kornelija Maciuliene, Grazina Drasutiene, Diana Ramasauskaite

**Affiliations:** 1 Lithuanian University of Health Sciences, Eiveniu str. 2, 50167 Kaunas, Lithuania; 2 Victoria Hospital, Kirkcaldy, Fife KY2 5AH Scotland, UK; 3 Vilnius Maternity Hospital, Tyzenhauzu str. 18A, 02106 Vilnius, Lithuania; 4 Vilnius University Hospital Santaros Klinikos, Santariskiu str. 2, 08661 Vilnius, Lithuania

**Keywords:** Caesarean section, Rate, Robson classification, Intervention, Nulliparas

## Abstract

**Background:**

To evaluate the role of the TGCS to reduce the caesarean section (CS) rate among nulliparas (Robson groups 1 and 2) and to find out which group of women have reduced the CS rate by using this tool.

**Methods:**

The Robson classification was introduced in Lithuanian hospitals prospectively classifying all the deliveries in 2012. The CS rate overall and in each Robson group was calculated and the results were discussed. The analysis was repeated in 2014 and the data from the selected hospitals were compared using MS EXCEL and SPSS 23.0.

**Results:**

Nulliparas accounted for 43% (3746/8718) and 44.6% (3585/8046) of all the deliveries in 2012 and 2014 years, respectively. The CS rate among nulliparas decreased from 23.9% (866/3626) in 2012 to 19.0% (665/3502) in 2014 (*p* < 0.001).The greatest decrease in absolute contribution to the overall CS rate was recorded in groups 1 (*p* = 0.005) and 2B (p < 0.001). Perinatal mortality was 3.5 in 2012 and 3.1 in 2014 per 1000 deliveries (*p* = 0.764).

**Conclusion:**

The TGCS can work as an audit intervention that could help to reduce the CS rate without a negative impact on perinatal mortality.

## Background

The caesarean section (CS) rate had been increasing for several decades worldwide [1]. In Lithuania it has increased from 9.49% in 1995 to 24.71% in 2010 [2]. Similar rates were reported in Canada (27%), the USA (33%), Brazil (50%) or Australia (31%) [1]. On the other hand, northern European countries, such as Finland (16%) or Norway (17%), are known for lower rates [1]. Caesarean section not only increases societal economic burden but also has huge impact on the future fertility potential of the woman. This has led to debate among obstetricians and gynaecologists, health care policy makers and service users to define strategies in order to reduce the rate of primary CSs. As a consequence, various strategies have been recommended to improve the antenatal and intrapartum care of women, to increase normal vaginal delivery rates, thus reducing the incidence of primary CS rates [3–7]. Most of these strategies are either quality improvement tools or audit and feedback processes [3]. One such approach, proposed by the World Health Organization [8], is to use the Robson classification (also known as the 10-group classification system (TGCS)) as an audit tool [9]. It helps to identify the groups of women who are responsible for the increasing CS rate the most (Table 1), so that corrective solutions could be put in place to reduce the CS rates.

**Table 1:** The 10-group classification system

No.	Group
Group 1	Nulliparous, single cephalic, 37 weeks, in spontaneous labour
Group 2A	Nulliparous, single cephalic, 37 weeks, induced labour
Group 2B	Nulliparous, single cephalic, 37 weeks, CS before labour
Group 3	Multiparous (excluding prev. CS), single cephalic, 37 weeks, in spontaneous labour
Group 4A	Multiparous (excluding prev. CS), single cephalic, 37 weeks, induced labour
Group 4B	Multiparous (excluding prev. CS), single cephalic, 37 weeks, CS before a labour
Group 5A	Previous CS, single cephalic, 37 weeks, induced labour
Group 5B	Previous CS, single cephalic, 37 weeks, CS before labour
Group 5C	Previous CS, single cephalic, 37 weeks, in spontaneous labour
Group 6	All nulliparous breeches
Group 7	All multiparous breeches (including prev. CS)
Group 8	All multiple pregnancies (including prev. CS)
Group 9	All abnormal lies (including prev. CS)
Group 10	All single cephalic, ≤ 36 weeks (including prev. CS)

It is well-known that nulliparous women are a special group in labour as they represent almost a half of all the deliveries. They have a greater risk of adverse outcomes during their delivery compared with multiparas [10]. On the other hand, the mode of delivery is of great importance, because it would influence on planning for a subsequent pregnancy and mode of delivery. It has been reported that women whose deliveries end by an operation have a risk of at least 50–60% of delivering by CS in a subsequent delivery [11]. Nulliparous women make up the first, the second and the sixth and might be a part of the eight, the ninth and the tenth groups according to the TGCS. But only groups 1 and 2 are not influenced by the number of foetuses, their presentation or gestation favourable to the caesarean section. Therefore, these important groups of patients should be studied carefully as regards conduct of labour and the interventions they had during labour.

The aim of this study was to evaluate the role of the implementation of the TGCS as a national audit intervention to reduce the CS rate among nulliparous women, with a singleton pregnancy, cephalic presentation at term pregnancy either in spontaneous or induced labour or had a planned elective CS (Robson groups 1 and 2). Our objective was to find out which group of women has reduced the CS rate by using the TGCS, which might be beneficial for other countries looking where to start from.

## Methods

This paper presents results of one part of a multifaceted intervention that was performed in 2012–2016 in Lithuania. This intervention consisted of three parts:

- the implementation of the Robson classification nationally (2012–2014),
- a national educational course (2015) and
- an audit performed in some randomly selected hospitals (2016).

The Robson classification has been used for this two phase study (Table 1).

Before the national interventional study, the Lithuanian obstetricians and gynaecologists were made aware of the usefulness of the Robson classification as a quality improvement tool. However it had not been formally endorsed nationally and was not used in everyday practice. In 2011, the Lithuanian Society of Obstetricians and Gynaecologists (LSOG) organised a special meeting to which obstetricians and gynaecologists and the authorities of all Lithuanian hospitals were invited. The Caesarean Section Working Group of the LSOG revisited the principles of the TGCS and encouraged the participants of the meeting to classify the deliveries routinely. All the participants were invited to join the study and to send relevant data to the study coordinators.

The study spanned over two periods. During the first period (from January 1 to December 31 of 2012), in the vast majority of Lithuanian hospitals all the deliveries were classified by their clinicians and summary data were sent to the study investigators. Continuous assistance was provided by study investigators where difficulties in classifying a woman arose. In 2013 all the data from the hospitals were analysed and a conference was organised specifically for this project. The attendees of the conference included administrators of the participating hospitals, members of the LSOG, the Lithuanian Health Ministry and the members of the Lithuanian Parliament. During the conference, the overall CS rates and the CS rates in different groups according to the TGCS were assessed and compared between different hospitals [12]. This has led to a conclusion that the CS rate overall and in different groups of women is higher than recommended [11] and can be lowered. No specific recommendations or interventions were proposed and no special groups of women were singled out. Another purpose of the conference was to send a message to all hospitals that the CS rates were important at national level and they would be analysed in the future.

During the second period (from January 1 to December 31 of 2014), the classification of deliveries according to the Robson classification was recorded and the data were sent to the study investigators.

At the time of the study, there were 33 hospitals providing obstetrical care in Lithuania. The inclusion criteria for hospitals were: that there should be at least 300 deliveries per year before national intervention was conducted and no other programs dedicated to reduce the CS rate had to be performed during the intervention. On the whole, 26 hospitals admitted these inclusion criteria.

The maternity hospitals within Lithuania are currently categorized as follows:

Level IIA hospitals, which provide health care services to women with low-risk pregnancies and deliveries. In 2012, there were 25 level IIA hospitals with 9554 deliveries. The inclusion criteria were met by 18 of them;

Level IIB hospitals, which provide health care services to women with low- and high- (not requiring tertiary-level care, for example, a preterm delivery after 34 gestational weeks) risk pregnancies and deliveries. At the beginning of the study, there were 6 level IIB hospitals with 11,966 deliveries;

Level III hospitals, which provide health care services to women with pregnancies and deliveries of all risk levels. In 2012, there were 2 level III hospitals with 7157 deliveries in Lithuania.

The necessary number of hospitals for inclusion in the study was calculated according to the number of deliveries in them. It had to be sufficient to represent the national data overall and in each stratum (level IIA, level IIB and level III hospitals). Therefore, three level IIA hospitals (with 1635 deliveries in total in 2012 and 1604 in 2014), one level IIB hospital (with 3205 deliveries in 2012 and 3075 in 2014) and one level III hospital (with 3878 deliveries in 2012 and 3367 in 2014 were selected randomly for inclusion in the study.

For this study, all the data on nulliparas (groups 1 and 2 according to the TGCS) were collected from the selected hospitals by a single investigator (JK). Where mistakes were found in the records, all the deliveries were checked and reclassified according to the TGCS principles. Socio-demographic and selected personal data (living area, marital status, education and age) were gathered using hospital delivery records and the national database of the Lithuanian Hygiene Institute. Specific delivery-related data were collected from the case notes. Some case notes that were absent, illegible or had missed information directly related to the delivery (i.e. the presence or absence of augmentation, cervical dilatation at the moment when the decision to perform a CS because of dystocia was made etc.) were not included in a specific CS’s analysis. Therefore, an imbalance between socio-demographic, personal and delivery-related data might appear.

### Outcomes

The primary outcome was the overall CS rate. Secondary outcomes were delivery-related data (indications for CS, cervical dilatation at the moment of oxytocin administration, cervical dilatation at the moment of the decision to perform a CS because of dystocia, and waiting time passed before the decision to perform a CS because of dystocia) and newborn-related outcomes (birth weight, Apgar scores and perinatal mortality). Also, potential risk factors were also assessed – socio-demographic data (living area, education, and marital status), personal data (age, maternal diseases (all diseases that are not related to pregnancy, i.e. eye, ear, pulmonary, cardiovascular, renal and other pathologies) as well as pregnancy-related conditions (i.e. gestational hypertensive disorders, gestational cholestasis, gestational diabetes etc.).

Based on the analysis of indications for CSs proposed by Robson et al. [13], *a new modified classification* of indications for CSs in nulliparas assigned to group 1 was developed. The main difference was that various conditions (i.e. umbilical cord prolapse after the spontaneous onset of labour, placental abruption etc.) were grouped within a separate group. For the vast majority of such deliveries CS is unavoidable. Therefore, no specific strategies can be created to manage such a delivery vaginally. The other reason for modifying the classification was different management of delivery itself: a different perception of latent and active phases, oxytocine dosage etc. A modified classification included whether variables such as augmentation with or without oxytocin, dilatation of the cervix (complete cervical dilatation (CCD) and incomplete cervical dilatation (< 10 cm) (ICD)), suspected fetal compromise (FC) and other conditions such as placental abruptions, umbilical cord prolapse, and elective CSs with the onset of spontaneous labour. The indications for CS in group 1 were classified as dystocia, FC and others.

### Participants

All nulliparous women with a single cephalic, term pregnancy in spontaneous (group 1) or induced labour (group 2A) or an elective CS (group 2B) who gave birth at the selected hospitals during the study period. Nulliparous women without spontaneous labour and falling within groups 2A or 2B formed group 2.

### Statistical analysis

The data were analysed using MS Excel and IBM SPSS Statistics 23.0 for Windows. There were 3 groups of hospitals which represented health care facilities providing obstetrical care of different levels. It was estimated that a minimum sample size of 1197 of all deliveries per each stratum would be needed to detect a 5% change in the CS rate with a power of 80% and a significance level of 5%. From these deliveries only nulliparous women falling within groups 1 and 2 according to the TGCS were selected and the outcomes of their deliveries in 2012 and 2014 were compared. The comparison of study groups was conducted using chi-squared test, and if assumptions for it were not met – the Fisher’s exact test.

## Results

There were 8718 and 8046 deliveries in 2012 and 2014, respectively. Nulliparas accounted for 43% (3746/8718) and 44.6% (3585/8046) of all the deliveries in those years, respectively. The socio-demographic and personal data are compared in Table 2. There were more women living in urban areas than in rural (*p* = 0.003) and married vs. single women (*p* < 0.001) in both years. The education, age and number of women with any maternal diseases did not differ between 2012 or 2014 (Table 2).

**Table 2:** Socio-demographic and personal data

	2012	2014	*p* value
Living area			
urban, n (%)	3117 (83.2)	2886 (80.5)	*p* = 0.003
rural, n (%)	629 (16.8)	699 (19.5)	
Education			
basic, primary and secondary, n (%)	1749 (46.7)	1649 (46.0)	*p* = 0.552
higher, n (%)	1997 (53.3)	1936 (54.0)	
Marital status			
single, n (%)	1326 (35.4)	1089 (30.4)	*p* < 0.001
married, n (%)	2420 (64.6)	2496 (69.6)	
Age			
< 20, n (%)	1264 (33.7)	1142 (31.9)	*p* = 0.198
20–34, n (%)	2270 (60.6)	2224 (62.0)	
≥ 35, n (%)	212 (5.7)	219 (6.1)	
Maternal diseases**,** n (%)	155 (4.1)	124 (3.5)	*p* = 0.129

There were 96.8% (3626/3746) case notes available for analysis in 2012, as compared to 97.7% in 2014 (3502/3585). The CS rate among nulliparas decreased from 23.9% (866/3626) in 2012 to 19.0% (665/3502) in 2014 (p < 0.001) (Table 3).The greatest decrease in absolute contribution to the overall CS rate was recorded in groups 1 (*p* = 0.005) and 2B (p < 0.001) (Table 3).

**Table 3:** The CS rate in 2012 and 2014

	Number of CS over total number of women in each group		Relative size of groups, %		CS rate in each group, %		p value	Absolute contribution to the оverall CS rate, %		p value
	2012	2014	2012	2014	2012	2014		2012	2014	
Group 1	426/2776	340/2698	76.6 2776/3626	77.0 2698/3502	15.3	12.6	*p* = 0.003	11.7 426/3626	9.7 340/3502	*p* = 0.005
Group 2	440/850	326/804	23.4 850/3626	23.0 804/3502	51.8	40.5	*p* < 0.001	12.1 440/3626	9.3 326/3502	*p* < 0.001
Group 2A	236/646	220/698	17.8 646/3626	19.9 698/3502	36.5	31.5	*p* = 0.052	6.5 236/3626	6.3 220/3502	*p* = 0.697
Group 2B	204/204	106/106	5.6 204/3626	3.0 106/3502	100	100	–	5.6 204/3626	3.0 106/3502	*p* < 0.001
Group 1 and 2	866/3626	666/3502			23.9	19.0	*p* < 0.001			

The greatest contribution to the overall CS rate in group 1 was made by dystocia in both years (Table 4). The majority of operations in this group were performed in patients, who had their labour augmented with oxytocin irrespective the complete cervical dilatation was reached or not (Table 4). The peculiarities of dystocia in group 1 are presented in Table 5. The greatest reduction in the contribution to the overall CS rate in group 1 was made by operations due to fetal compromise (*p* = 0.002) (Table 4).

**Table 4:** Indications for CS among women in group 1

Indication	Absolute contribution to the CS rate in group 1, %, 2012	Absolute contribution to the CS rate in group 1, %, 2014	p value	Absolute contribution to the overall CS rate, %, 2012	Absolute contribution to the overall CS rate, %, 2014	p value
FC (without oxytocin)	22.1 94/426	15.9 54/340	*p* = 0.032	2.6 94/3626	1.5 54/3502	*p* = 0.002
Dystocia	70.4 300/426	74.1 252/340	*p* = 0.258	8.3 300/3626	7.2 252/3502	*p* = 0.089
CCD without oxytocin	11.7 50/426	10.0 34/340	*p* = 0.447	1.4 50/3626	1.0 34/3502	*p* = 0.11
CCD with oxytocin	17.6 75/426	20.0 68/340	*p* = 0.401	2.1 75/3626	2.9 68/3502	*p* = 0.704
ICD without oxytocin	7.5 32/426	6.2 21/340	*p* = 0.472	0.9 32/3626	0.6 21/3502	*p* = 0.165
ICD with oxytocin	22.5 96/426	27.9 95/340	*p* = 0.085	2.6 96/3626	2.7 95/3502	*p* = 0.865
FC with oxytocin^a^	11 47/426	10.0 34/340	*p* = 0.646	1.3 47/3626	1.0 34/3502	*p* = 0.194
Others	7.5 32/426	10.0 34/340	*p* = 0.222	0.9 32/3626	1.0 34/3502	p = 0.697
Total				11.7 426/3626	9.7 340/3502	*p* = 0.006

^a^“FC with oxytocin” was attributed to “dystocia” because the reason why oxytocin was administered was dystocia

**Table 5:** Peculiarities of deliveries when the indication for CS is “dystocia”

	2012	2014	p value
Dystocia: CCD with oxytocin			
Cervical dilatation at the moment of oxytocin administration, cm (mean, SD)	4.69 (2.11)	5.7 (2.21)	0.007
Waiting time passed before making the decision to perform a CS because of dystocia, min (mean, SD)	88.3 (31.55)	87.8 (38.7)	0.933
Dystocia: ICD with oxytocin			
Cervical dilatation at the moment of oxytocin administration, cm (mean, SD)	3.97 (2.16)	4.1 (2.24)	0.68
Cervical dilatation at the moment of the decision to perform a CS because of dystocia, cm (mean, SD)	5.39 (2.53)	5.38 (2.61)	0.991
Waiting time passed before the decision to perform CS because of dystocia, min (mean, SD)	171.3 (125.7)	217.21 (164.47)	0.033

The number of CSs in group 2 decreased from 51.8% (440/850) in 2012 to 40.5% (326/804) in 2014. The greatest decrease was seen in group 2B. The vast majority of them were performed because of maternal diseases, post-term pregnancy and fetus related conditions (Table 6).

**Table 6:** Indications for operations in women with elective CSs

Indication	Absolute contribution to the CS rate in group 2B, %, 2012	Absolute contribution to the CS rate in group 2B, %, 2014	p value	Absolute contribution to the overall CS rate, %, 2012	Absolute contribution to the overall CS rate, %, 2014	p value
Placental and umbilical cord pathology	9.8 20/204	10.4 11/106	*p* = 0.873	0.6 20/3626	0.3 11/3502	*p* = 0.129
Fetus related conditions	16.2 33/204	11.3 12/106	p = 0.25	0.9 33/3626	0.3 12/3502	*p* = 0.003
Maternal diseases	48.0 96/204	49.0 52/106	*p* = 0.741	2.7 96/3626	1.5 52/3502	*p* < 0.001
Suspected fetal macrosomia	6.9 14/204	9.4 10/106	*p* = 0.424	0.4 14/3626	0.3 10/3502	*p* = 0.465
Preeclampsia	3.4 7/204	4.7 5/106	*p* = 0.575	0.2 7/3626	0.1 5/3502	*p* = 0.603
Post-term pregnancy	16.7 34/204	15.1 16/106	*p* = 0.719	0.9 34/3626	0.5 16/3502	*p* = 0.015
Total				5.6 204/3626	3.0 106/3502	*p* < 0.001

The study did not reveal any change in perinatal mortality (*p* = 0.764). Among 3746 deliveries in 2012 and 3585 in 2014, it was 3.5 and 3.1 per 1000 deliveries, respectively (Table 7). The Apgar score after 5 min changed in 2014 (*p* = 0.003) mostly because of a decreased number of newborns with the Apgar score ≥ 9 points and an increased number of newborns with the Apgar score of 7–8 points.

**Table 7:** Newborn-related outcomes

Indicator	2012	2014	p value
Newborn weight			
< 3000 g, n (%)	476 (12.7)	429 (12.0)	*p* = 0.482
3000–3999 g, n (%)	2809 (75.0)	2691 (75.1)	
≥ 4000 g, n (%)	461 (12.3)	465 (13.0)	
Apgar score after 5 min			
< 7, n (%)	6 (0.2)	2 (0.1)	*p* = 0.003
7–8, n (%)	141 (3.8)	188 (5.2)	
≥ 9, n (%)	3599 (96.1)	3395 (94.7)	
Perinatal mortality			
antepartum deaths, n	10	7	*p* = 0.764
intrapartum deaths, n	1	0	
not defined, n	–	–	
early neonatal deaths, n	2	4	
perinatal mortality	3.5/1000	3.1/1000	*p* = 0.764

## Discussion

This study has shown that the implementation of the Robson classification has had a positive impact on the reduction of the CS rate among nulliparas in Lithuania. After the intervention, this rate has decreased by 4.9 pp. The greatest decrease was recorded in group 2B (*p* < 0.001) and group 1 (*p* = 0.005). Moreover, the highest reduction in the CS rate among nulliparas with spontaneous labour was due to the indication “fetal compromise” (*p* = 0.002). Despite the fact that the CS rate decreased, the perinatal morbidity did not change. A statistically significant difference in Apgar score in 5 min can be considered as clinically insignificant – the observed difference was mainly due to large sample size. A reduction of the CS rate after the implementation of the TGCS in clinical practice has been reported in other studies too [13–17]. Scarella et al. reported a 7% reduction (from 32.2 to 25.2%) in CSs among nulliparas who were assigned to group 1 or 2 in one Chilean regional health centre [14]. Despite the fact that this reduction was greater than the one recorded in the present study, it is important to mention that the starting rate in Chile was almost 10% higher, and after the intervention is still 6% higher, than the one recorded in our study. A reduction of more than 20% was reported in a study from Brazil that was performed at one university hospital [17]. According to the authors of that study, the CS rate in groups 1 and 2 decreased from 34.6 to 13.5% in a 10-month period following the implementation of the Robson classification [17]. This is more than 4 times higher than in our study and could be explained by a very high initial CS rate. Svelato et al. and Blomberg have also reported a reduction in the CS rate after starting to use the TGCS [6, 15]. Yet, the interventions in their studies were multifactorial. They included not only the implementation of the Robson classification, but also the management of labour, interpretation of the cardiotocogram (CTG) etc. [6, 15]. Svelato et al. analysed Robson groups 1–4 in one hospital in Italy. They reported a 6.9 pp. (17.2% to 10.3%) decrease in the CS rate in these groups after the intervention [15]. The target group of Blomberg consisted of nulliparas falling within group 1 according to the TGCS, who delivered in one tertiary level hospital in Sweden [6]. The CS rate in group 1 decreased from 10 to 3% after the study [6]. In addition to this, the CS rate in Robson groups 1 and 2 decreased by 10.5 pp. (from 17.5 to 7%), i.e. twice the decrease reported in our study [6]. It is well known that the CS rates in the Northern European countries are among the lowest [1]. This might be influenced by organizational and cultural differences in the management of deliveries. Despite the fact that the latter two studies describe multifactorial interventions, the impact of the Robson classification cannot be downplayed because the Hawthorne effect might be attributed to it [18]. This effect describes a change in an individual’s behaviour after finding out about being observed. It was first noticed when performing experiments in one electricity company [18]. But it is still relevant nowadays and can explain why implementation of the TGCS is enough to work as an audit system and reduce the CS rates [12, 14].

The analysis in this study revealed that the number of CSs decreased the most after the reconsideration of the indications for elective CSs. A major change in the CS rate in group 2B owes to the reduction of the indication “post-term pregnancy”. This could be explained by the fact that hospitals could postpone induction of the delivery or elective CS and wait for a spontaneous onset of labour. The other reason for CS is maternal diseases. Despite the fact the number of women with identifiable maternal diseases did not differ statistically significantly between 2012 and 2014, fewer women were operated because of conditions related to eyes, ears, heart pathologies, previous cervical conisation etc. This means that indications for elective operative deliveries should be stricter in all hospitals and all cases should be discussed in a multi-disciplinary setting in case of uncertainty. The other reason for the reduced number of women in this group might be that some deliveries can be induced rather than ended by an elective CS. The relative size of group 2A increased in 2014, although without statistical significance. On the other hand, the CS rate in this group decreased. This might show that a greater number of induced deliveries were managed successfully.

The decrease in the CS rate among nulliparas with spontaneous labour owes mainly to FC managed without oxytocin. This might be explained by two reasons. The first one is that the initial numbers were very high. Our study has revealed that these numbers were more than double the one reported by Robson et al. [12]. The other reason that could explain a significant decrease in CSs in this group is better evaluation of the CTG resulting from the Hawthorne effect. It is possible that changes in the CTG in a woman without oxytocin were attributed to insignificant changes and this was different as it was prior to the intervention phase. It is important to emphasize that newborn-related data and perinatal mortality did not change as our sample size was not calculated to assess these outcome parameters.

The vast majority of operations among nulliparas with spontaneous labour were performed because of dystocia. This outcome correlates with other studies [12]. A more detailed analysis has revealed that operations in this group were mostly performed on women who were treated with oxytocin and irrespective of whether they reached a full dilatation of the cervix or not. A high number of operations in women with full cervical dilatation could be explained by an insufficient waiting time for delivery of the fetus. According to the national guidelines that were developed based on international recommendations, a normal duration of the second stage of delivery in nulliparas is about 2 h [19]. In case of epidural analgesia it is about 3 h [19]. Our study has shown that the mean duration of waiting is approximately 1.5 h. This duration is less than recommended in the national guideline. Therefore, if the number of women in this group increase but the length of second stage is not adhered to national guidance, then an increased number of women will have operative deliveries in the future too.

### Strengths and limitations

The strengths of the study are the following: all the data were checked and gathered by the same single study investigator, JK. This have led to an equal classification of all the deliveries; the study represents national data; this study introduces a new classification (based on Robson proposed classification of indications for CSs of intrapartum caesarean sections. This enables us to analyse and compare not only the number of CSs but also the reasons for them.

The limitation of the study is that not all case notes were available for analysis and no maternal outcomes are analysed and discussed.

## Conclusions

This two phase study in Lithuania has demonstrated that TGCS can work as an audit intervention that could help to reduce the CS rate without a negative influence on perinatal mortality. This system had the greatest impact on nulliparas for whom an elective CS was planned or those with suspected fetal compromise.

## Abbreviations

CCD: Complete cervical dilatation
CS: Caesarean section
CTG: Cardiotocogram
FC: Fetal compromise
ICD: Incomplete cervical dilatation
LSOG: Lithuanian society of obstetricians and gynecologists
TGCS: The 10-group classification system
